# Supplementary material for: The Temporal Dynamics of Sensitivity, Aflatoxin Production, and Oxidative Stress of Aspergillus flavus in Response to Cinnamaldehyde Vapor
Source: Foods. 2023 Nov 29;12(23):4311. doi: 10.3390/foods12234311 (PMC10706189; doi:10.3390/foods12234311)
Supplement: Supplementary file 1 [file foods-12-04311-s001.zip › foods-2708139-supplementary.pdf]

SUPPLEMENTARY MATERIAL

The Temporal Dynamics of Sensitivity, Aflatoxin Production, and Oxidative Stress of *Aspergillus flavus* in Response to Cinnamaldehyde Vapor

Ajuan Niu, Leilei Tan, Song Tan, Guangyu Wang* and Weifen Qiu

Key Laboratory of Grains and Oils Quality Control and Processing, Collaborative Innovation Center for Modern Grain Circulation and Safety, College of Food Science and Engineering, Nanjing University of Finance and Economics, Nanjing 210023, China; tss1054530271@163.com (S.T.); weifenqiu711@163.com (W.Q.)

* Correspondence: gywang@nufe.edu.cn

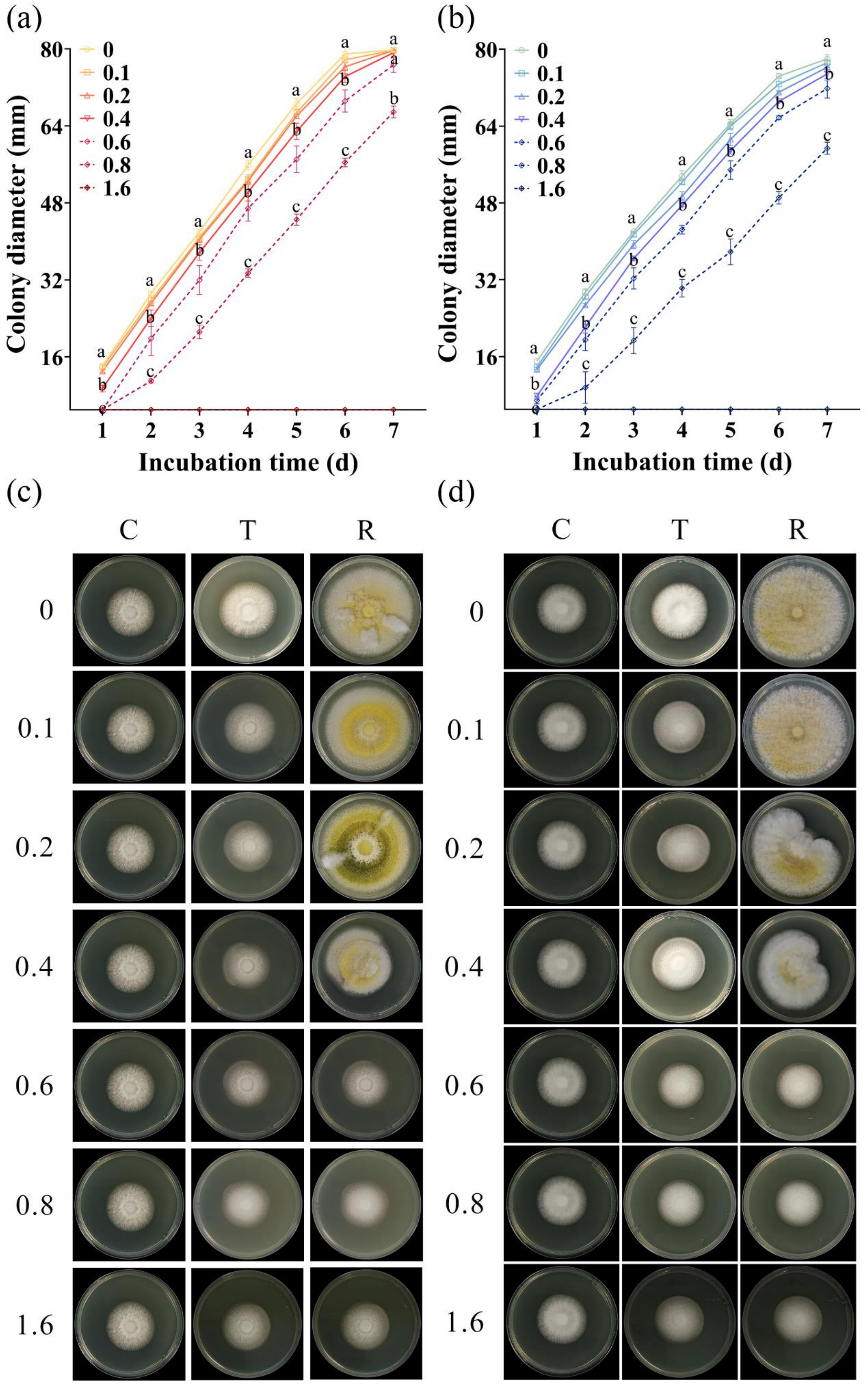

Figure S1. CA vapor sensitivity pattern of *A. flavus*. Growth curves (a, b) and

morphological changes (c, d) of aflatoxin-producing *A. flavus* JX-4 and aflatoxin-non-producing *A. flavus* JX-1. C, control group, T, group treated with CA vapor for 12 h, R, group that CA vapor disappeared after 12 h treatment. 0, 0.1, 0.2, 0.4, 0.6, 0.8 and 1.6 were the concentrations of CA vapor. The colony diameters of spores treated by 0, 0.4 and 0.8 μL/mL CA vapor were selected as typical representatives. Values in the same incubation time with different lowercase letters are significantly different ($p < 0.05$).

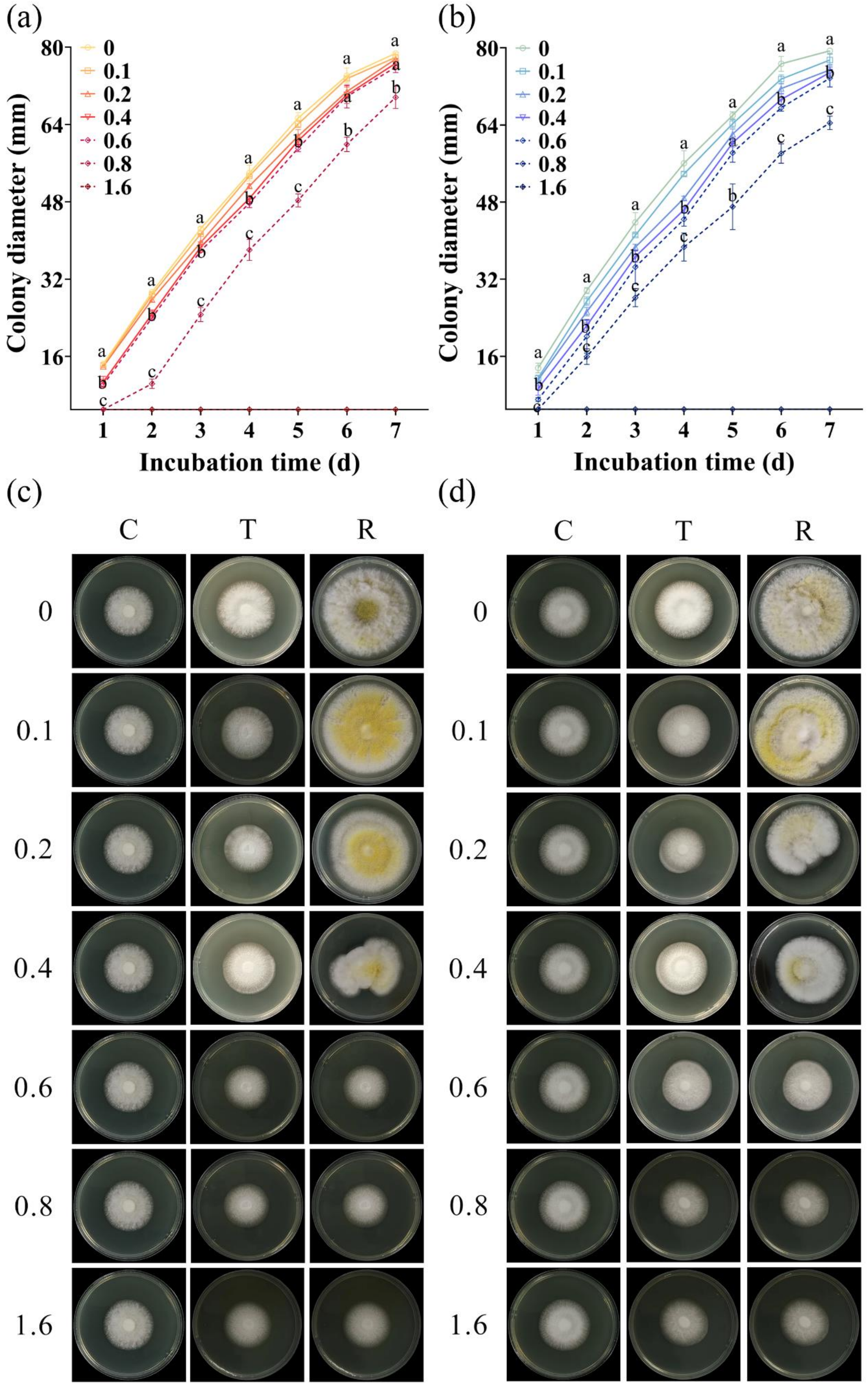

Figure S2. CA vapor sensitivity pattern of *A. flavus*. Growth curves (a, b) and

morphological changes (c, d) of aflatoxin-producing *A. flavus* HN-8 and aflatoxin-non-producing *A. flavus* JX-2. C, control group, T, group treated with CA vapor for 12 h, R, group that CA vapor disappeared after 12 h treatment. 0, 0.1, 0.2, 0.4, 0.6, 0.8 and 1.6 were the concentrations of CA vapor. The colony diameters of spores treated by 0, 0.4 and 0.8 μL/mL CA vapor were selected as typical representatives. Values in the same incubation time with different lowercase letters are significantly different ($p < 0.05$).

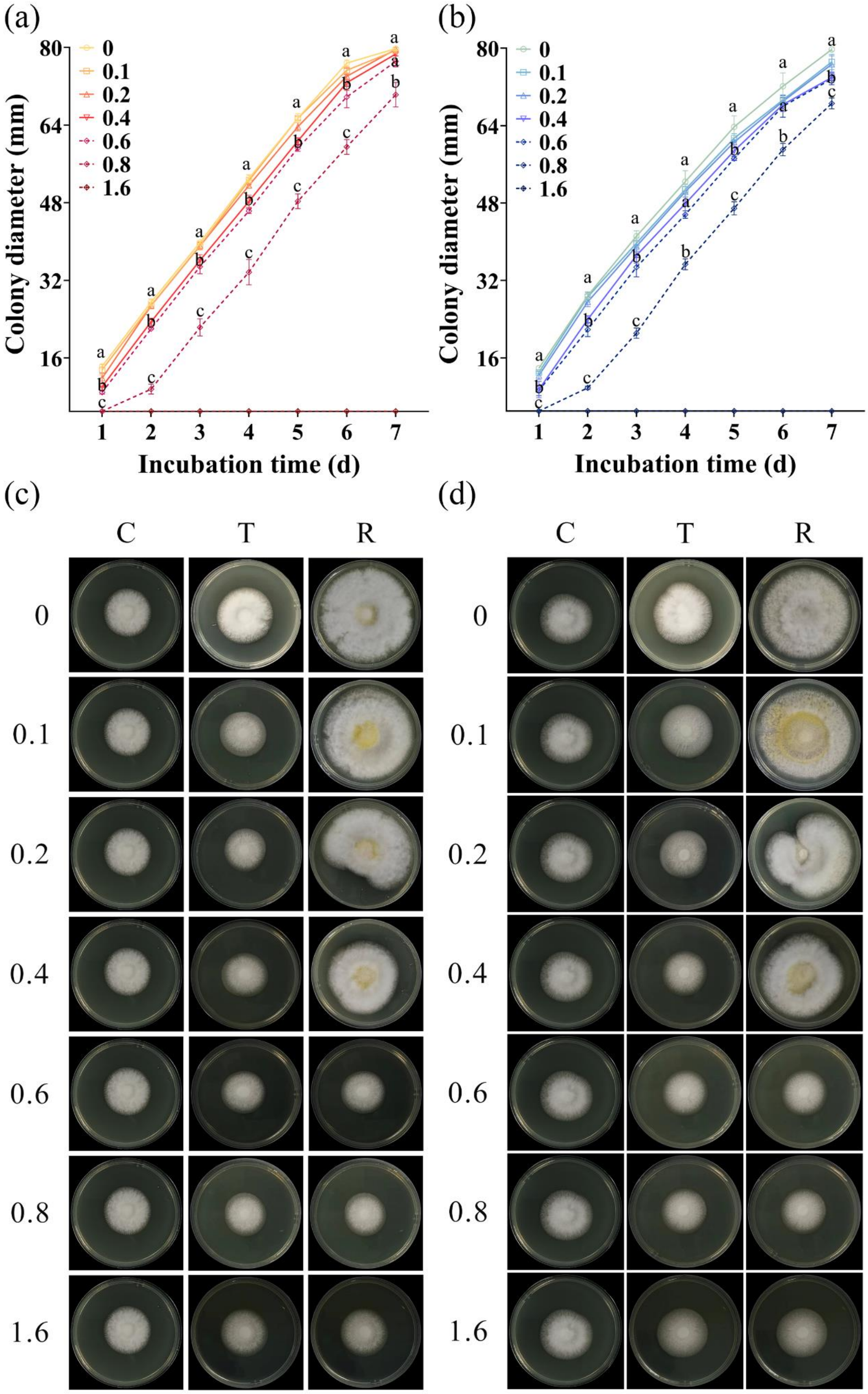

Figure S3. CA vapor sensitivity pattern of *A. flavus*. Growth curves (a, b) and

morphological changes (c, d) of aflatoxin-producing *A. flavus* FJ-5 and aflatoxin-non-producing *A. flavus* FJ-4. C, control group, T, group treated with CA vapor for 12 h, R, group that CA vapor disappeared after 12 h treatment. 0, 0.1, 0.2, 0.4, 0.6, 0.8 and 1.6 were the concentrations of CA vapor. The colony diameters of spores treated by 0, 0.4 and 0.8 μL/mL CA vapor were selected as typical representatives. Values in the same incubation time with different lowercase letters are significantly different ($p < 0.05$).

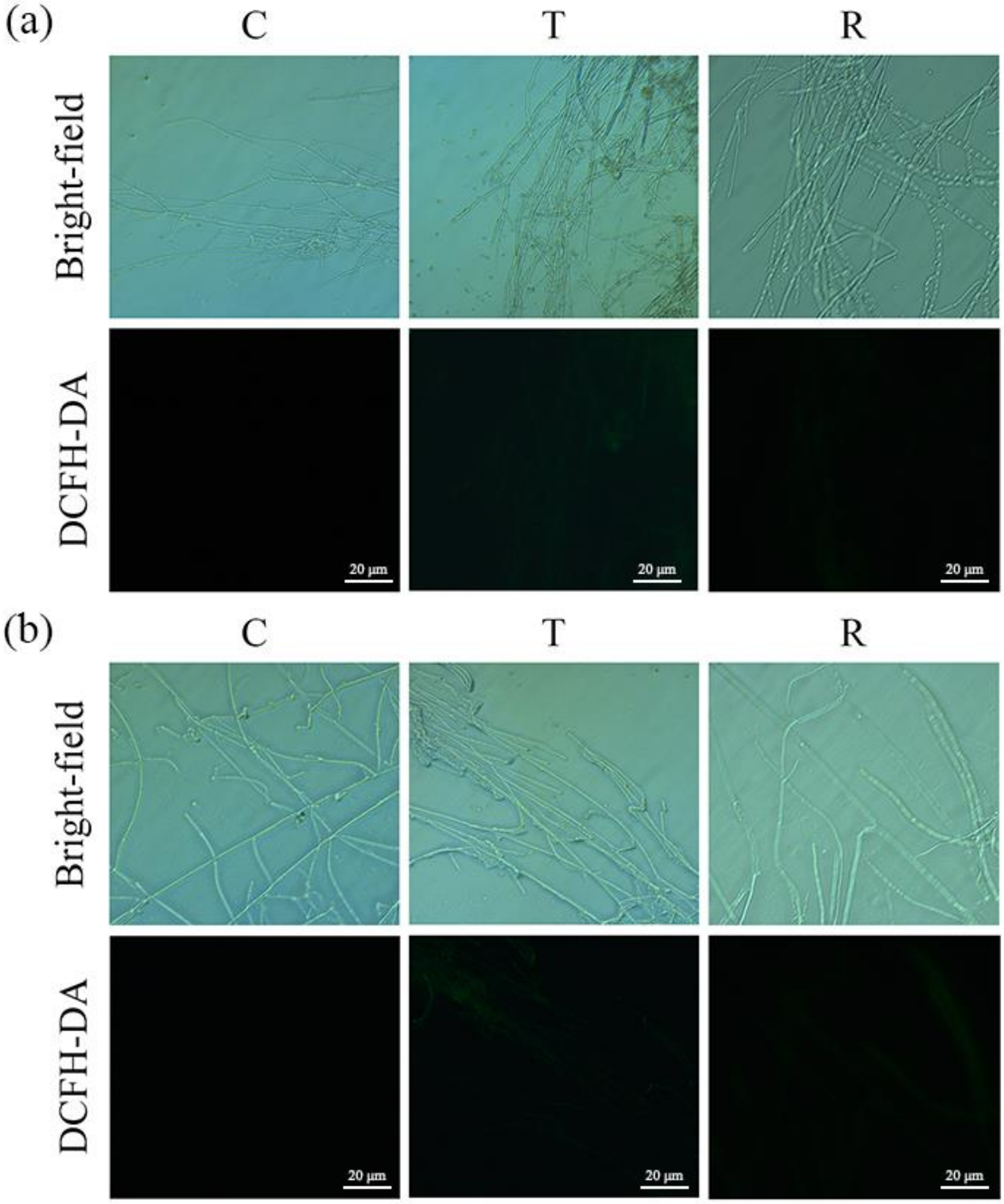

Figure S4. Changes of ROS in *A.flavus* JX-4 (a) and *A. flavus* JX-1 (b). C, control group, T, group treated with CA vapor for 12 h, R, group that CA vapor disappeared after 12 h treatment.

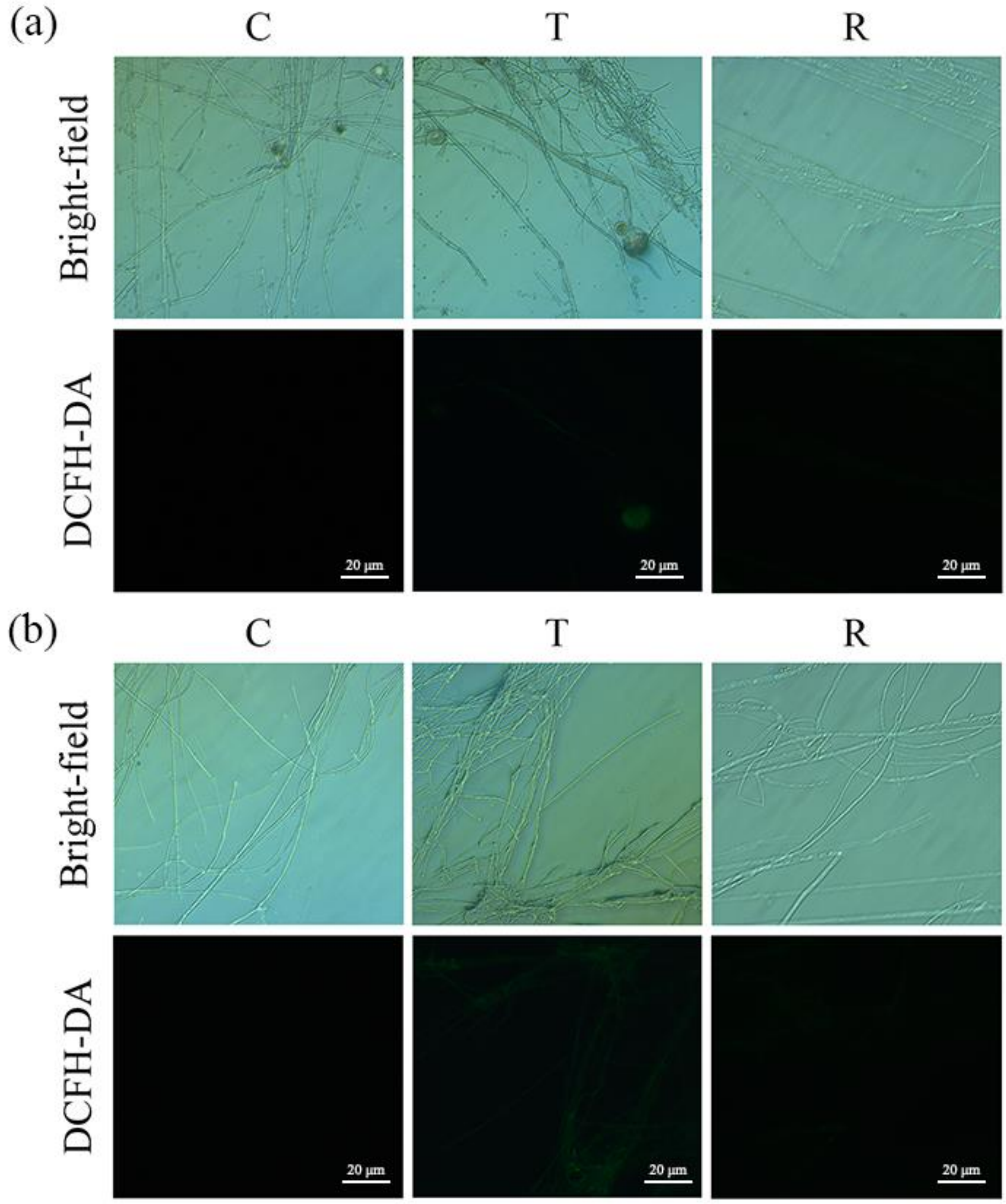

Figure S5. Changes of ROS in *A.flavus* HN-8 (a) and *A. flavus* JX-2 (b). C, control group, T, group treated with CA vapor for 12 h, R, group that CA vapor disappeared after 12 h treatment.

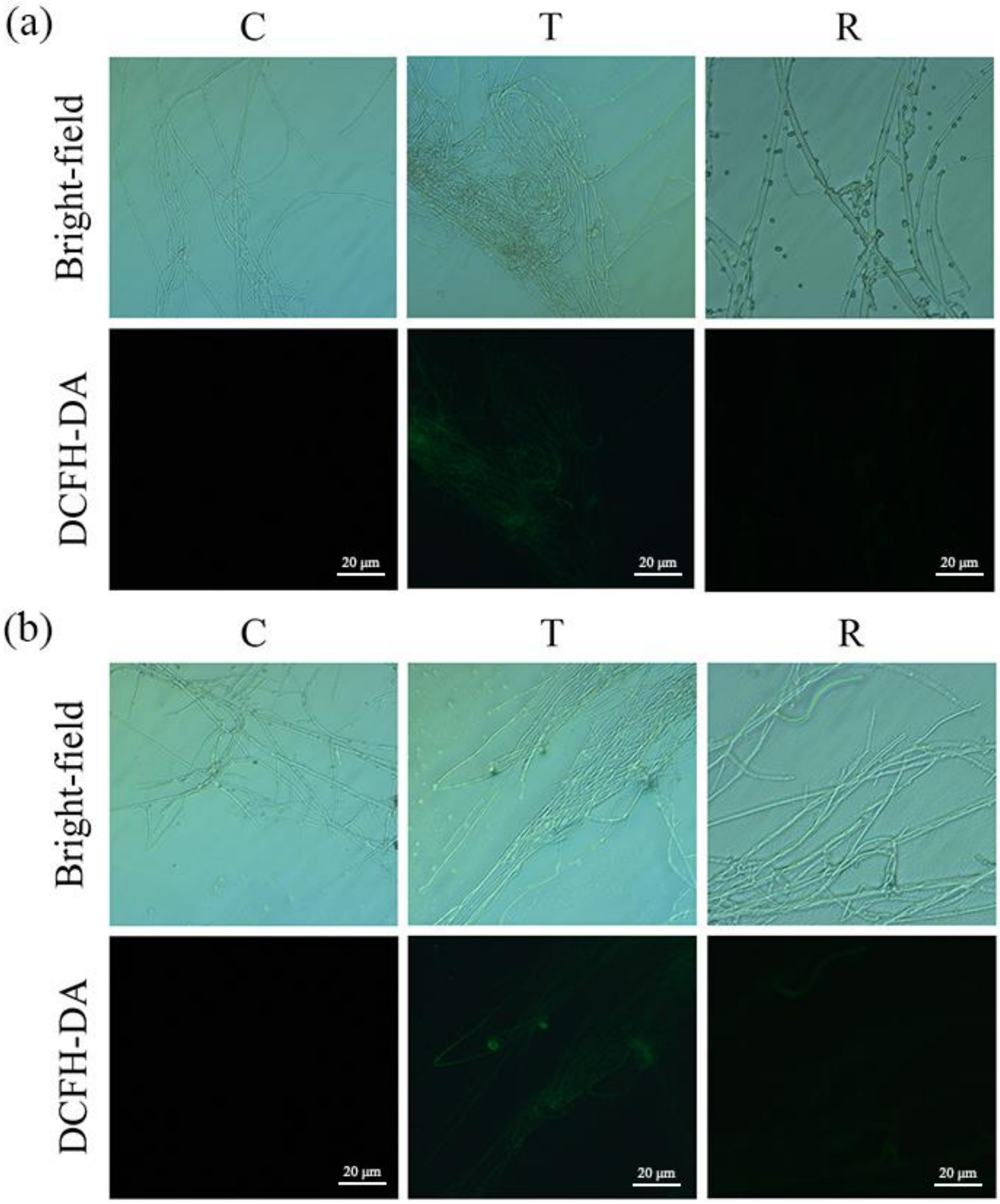

Figure S6. Changes of ROS in *A.flavus* FJ-5 (a) and *A. flavus* FJ-4 (b). C, control group, T, group treated with CA vapor for 12 h, R, group that CA vapor disappeared after 12h treatment.

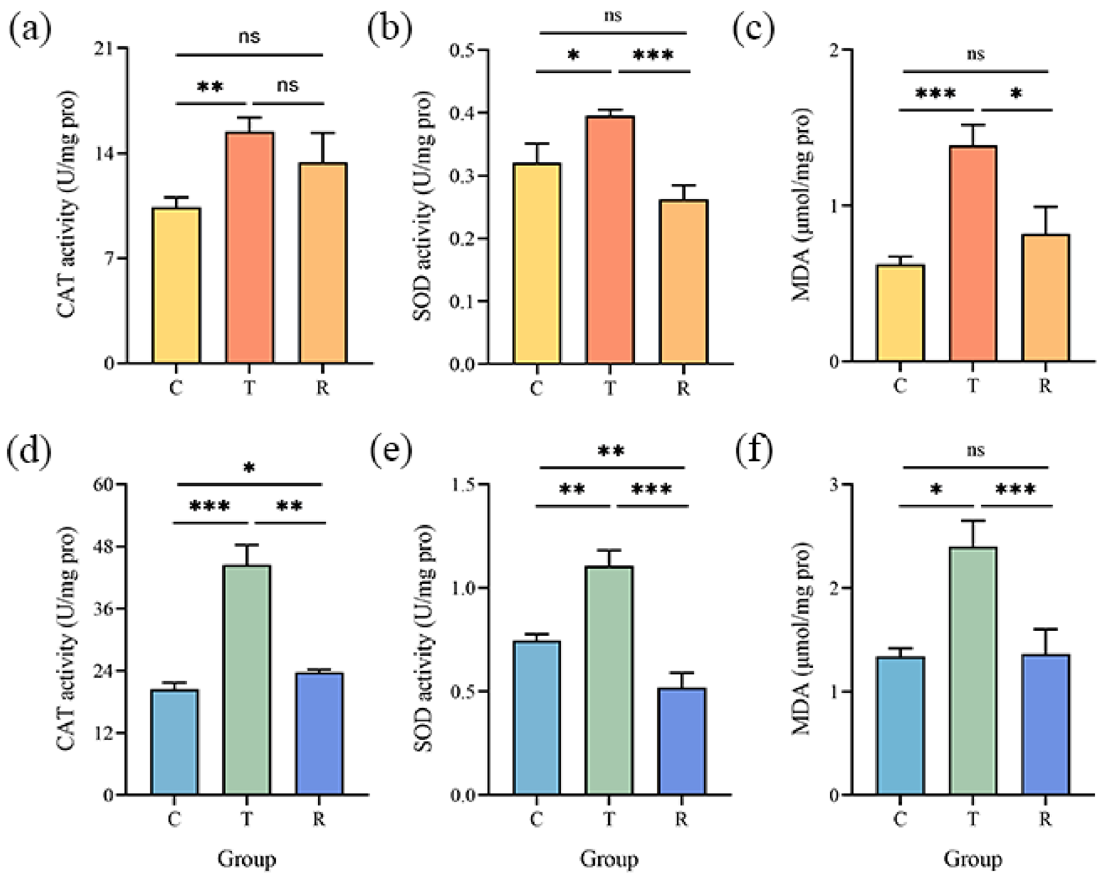

Figure S7. Changes of CAT (a, d), SOD (b, e) activities and MDA content (c, f) in *A. flavus* JX-4 (upper panel) and *A. flavus* JX-1 (lower panel). ns, no significant differences, *, $p < 0.05$, **, $p < 0.01$, ***, $p < 0.001$. C, control group, T, group treated with CA vapor for 12 h, R, group that CA vapor disappeared after 12 h treatment.

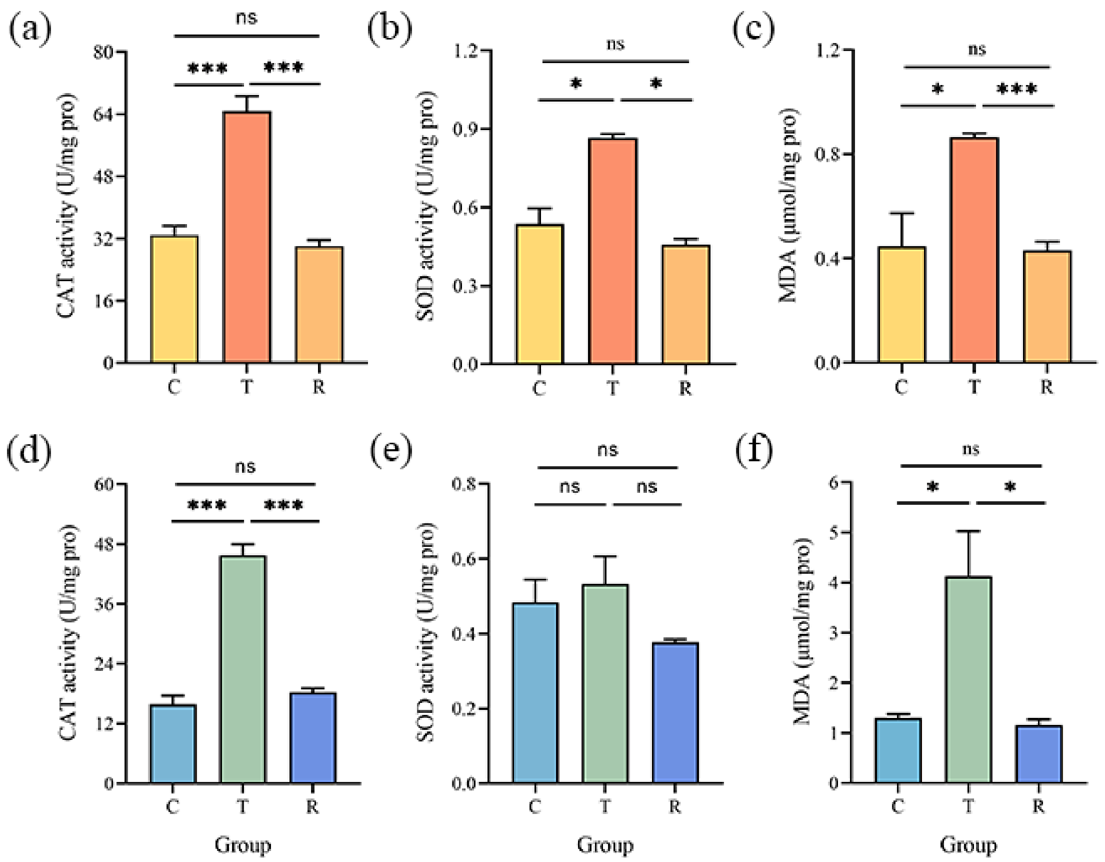

Figure S8. Changes of CAT (a, d), SOD (b, e) activities and MDA content (c, f) in *A. flavus* HN-8 (upper panel) and *A. flavus* JX-2 (lower panel). ns, no significant differences, *, $p < 0.05$, **, $p < 0.01$, ***, $p < 0.001$. C, control group, T, group treated with CA vapor for 12 h, R, group that CA vapor disappeared after 12 h treatment.

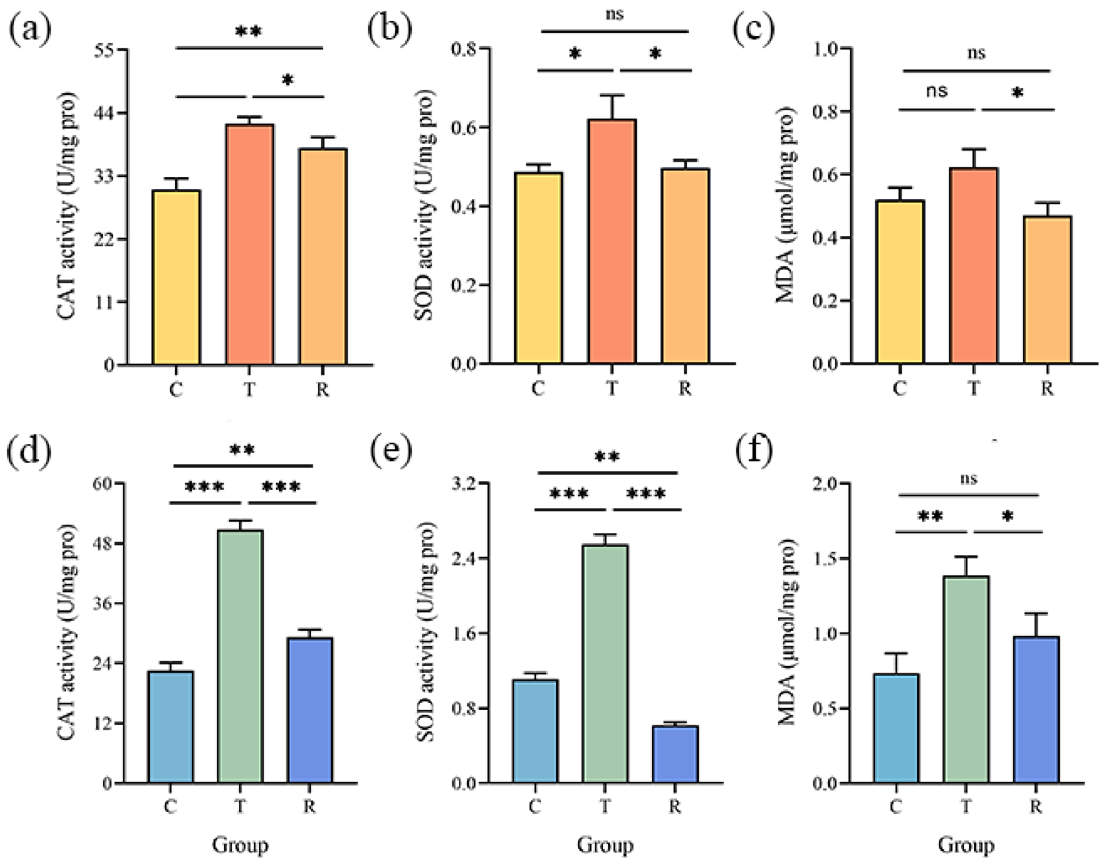

Figure S9. Changes of CAT (a, d), SOD (b, e) activities and MDA content (c, f) in *A. flavus* FJ-5 (upper panel) and *A. flavus* FJ-4 (lower panel). ns, no significant differences, *, p < 0.05, **, p < 0.01, ***, p < 0.001. C, control group, T, group treated with CA vapor for 12 h, R, group that CA vapor disappeared after 12 h treatment.
